# The Italian Diabetes and Exercise Study 2 (IDES-2): a long-term behavioral intervention for adoption and maintenance of a physically active lifestyle

**DOI:** 10.1186/s13063-015-1088-0

**Published:** 2015-12-11

**Authors:** Stefano Balducci, Massimo Sacchetti, Jonida Haxhi, Giorgio Orlando, Silvano Zanuso, Patrizia Cardelli, Stefano Cavallo, Valeria D’Errico, Maria Cristina Ribaudo, Nicolina Di Biase, Laura Salvi, Martina Vitale, Lucilla Bollanti, Francesco G. Conti, Antonio Nicolucci, Giuseppe Pugliese, for the Italian Diabetes and Exercise Study 2 (IDES_2) Investigators

**Affiliations:** Department of Clinical and Molecular Medicine, “La Sapienza” University of Rome, Via di Grottarossa, 1035-1039 - 00189, Rome, Italy; Diabetes Unit, Sant’Andrea Hospital, Rome, Italy; Metabolic Fitness Association, Monterotondo, Rome, Italy; Department of Human Movement and Sport Sciences, “Foro Italico” University, Rome, Italy; School of Science, University of Greenwich, London, UK; Laboratory of Clinical Chemistry, Sant’Andrea Hospital, Rome, Italy; Diabetes Unit, Fatebenefratelli San Pietro Hospital, Rome, Italy; Center for Outcomes Research and Clinical Epidemiology (CORE), Pescara, Italy

**Keywords:** Type 2 diabetes, Physical activity, Randomized controlled trial, Behavior change, Accelerometer

## Abstract

**Background:**

Physical activity (PA)/exercise have become an integral part of the management of type 2 diabetes mellitus (T2DM). However, current guidelines are difficult to put into action in this population due to a number of barriers, especially the lack of acceptable, feasible, and validated behavioral intervention strategies. The present manuscript reports the rationale, study design and methods, and design considerations of the Italian Diabetes and Exercise Study (IDES)-2, a randomized controlled trial testing the efficacy of a behavior change strategy in increasing total daily PA and reducing sedentary time (SED-time) in patients with T2DM.

**Methods/Design:**

Starting 7 January 2014, the IDES_2 began enrolling 300 patients with known T2DM of at least 1-year duration in three tertiary referral outpatient Diabetes Clinics in Rome. Additional requirements are age 40 to 80 years, body mass index 27 to 40 kg/m^2^, sedentary lifestyle, and physically inactive for at least 6 months, ability to walk 1.6 km without assistance, and eligibility after cardiovascular evaluation. Patients are randomized by center and within each center, by age and type of diabetes treatment to either the intervention or the control group. Patients in the intervention (INT) group (*n* = 150) receive theoretical and practical exercise counseling consisting of aggregated behavior change techniques (one individual theoretical counseling session plus eight twice-a-week individual theoretical and practical exercise counseling sessions) once a year for 3 years. Patients in the control (CON) group (*n* = 150), receive standard care, including general physician recommendations for daily PA. The primary outcomes are total daily PA and SED-time, as measured objectively by the use of an accelerometer. Secondary outcomes include physical fitness, modifiable cardiovascular risk factors, musculoskeletal disturbances, well-being/depression, and health-related quality of life.

**Discussion:**

The behavioral intervention strategy tested in the IDES_2 is based on solid theoretical grounds and uses several behavioral change techniques, two factors which were found to improve effectiveness of behavioral intervention. In addition, physicians and exercise specialists have been specifically trained for counselling/prescribing and supervising PA/exercise, respectively, in subjects suffering from metabolic disorders. Finally, the large sample size, the long study duration, and the objective measurement of PA allow statistically significant and scientifically robust conclusions to be drawn on the feasibility and efficacy of this intervention in T2DM patients.

**Trial registration:**

ClinicalTrials.gov; NCT01600937; 10 October 2012.

**Electronic supplementary material:**

The online version of this article (doi:10.1186/s13063-015-1088-0) contains supplementary material, which is available to authorized users.

## Background

Approximately 592 million people worldwide are predicted to be diagnosed with type 2 diabetes mellitus (T2DM) by the year 2035 [1]. This epidemic of T2DM is causally related to the rising incidence of obesity [2], with the increased calorie consumption combined with reduced energy expenditure contributing to the development of both conditions [3]. Recent data from the National Health and Nutrition Examination Survey (NHANES) highlight that the dramatic increase of obesity levels among US adults in the general population is mainly due to physical inactivity rather than to caloric intake [4]. In fact, another report from the NHANES indicates that less than 50 % of US adults achieve the recommended level of physical activity (PA) and ~30 % of them engage in no PA at all, though projections suggest an improvement [5]. The problem of sedentary behavior as well as of physical inactivity is even greater among people with T2DM or those at highest risk for developing this condition, who are well below national norms for PA [6]. Therefore, PA is now considered, together with diet and medication, as a cornerstone of T2DM management [7–9].

The joint position statement of the American College of Sports Medicine (ACSM) and the American Diabetes Association (ADA) [10] recommends that individuals with T2DM perform at least 150 minutes/week of moderate-to-vigorous aerobic exercise, plus moderate-to-vigorous resistance training at least 2–3 days/week. In addition, the ACSM/ADA guidelines recommend that individuals train under the supervision of qualified exercise trainers, particularly when undertaking resistance exercise, to ensure optimal health benefits and to minimize injuries; individuals are also encouraged to increase total daily unstructured (commuting, occupational, home, and leisure time [LT]) PA. These recommendations are based on a large body of experimental evidence which has been accumulating during the last decade. Two randomized controlled trials (RCTs), the Diabetes Aerobic and Resistance Exercise (DARE) study [11] and the Health benefits of Aerobic and Resistance Training in individuals with type 2 Diabetes (HART-D) study [12], have demonstrated that combined aerobic and resistance training is more effective than either one alone in reducing hemoglobin (Hb) A_1c_ in patients with type 2 diabetes. A systematic review and meta-analysis including the DARE and HART-D studies showed that structured exercise training, either aerobic, resistance, or both, is associated with HbA_1c_ reduction in patients with type 2 diabetes, especially if more than 150 minutes/week is undertaken and when combined with dietary advice [13]. Furthermore, another RCT, the Italian Diabetes and Exercise Study (IDES), showed that a strategy combining a supervised mixed (aerobic and resistance) exercise training program with structured exercise counseling was more effective than counseling alone in improving physical fitness and quality of life (QoL), ameliorating HbA_1c_ and other modifiable cardiovascular risk factors, reducing coronary heart disease (CHD) 10-year risk scores, and decreasing the number and/or dosage of medications, in a large cohort of sedentary subjects with T2DM [14–16]. Finally, recent findings suggest that higher sedentary time (SED-time) in T2DM individuals is associated with higher metabolic risk, independently of measured confounders and time spent in moderate-to-vigorous PA (MVPA) [17, 18], thus supporting the hypothesis that the biological responses to SED-time likely influence metabolic risk through pathways distinct from those of MVPA [19, 20].

However, it is difficult for people with T2DM to put into action these exercise recommendations for a number of external and internal barriers [21]. Data from trials for T2DM prevention [22, 23] and treatment [24] suggest the need to identify effective strategies to promote an adequate amount of PA in sedentary subjects such as those suffering from metabolic disorders. Unfortunately, available studies have applied PA-targeted behavioral interventions that are either heterogeneous or insufficiently detailed [25]. Moreover, these studies generally involved small samples, and changes in PA were not objectively measured. In the IDES, supervised training, in addition to providing significant health benefits, was successful also in promoting PA outside the supervised sessions, likely by improving patient’s knowledge, confidence, and sureness in his/her ability to perform PA effectively and safely on his/her own.

The IDES_2 is an open-label, parallel RCT aimed at assessing the efficacy of a behavioral intervention strategy derived from the IDES protocol in increasing total daily PA and reducing SED-time in patients with T2DM. This strategy combines the seven-step theoretical exercise counseling [26] provided to all IDES participants [27] with eight bi-weekly sessions, supervised by an exercise specialist in a dedicated gym facility. These sessions, which in the IDES were administered to the intervention group for the entire 12-month study duration [27], serve as a theoretical and practical counseling to promote and maintain a physically active behavior. Intervention is repeated every 12 months for 3 years, and PA is objectively monitored throughout the study by the use of an accelerometer. This intervention strategy is compared with current approach, that is, standard medical care including general physician recommendations on PA.

## Methods/Design

### Ethics

**Fig. 1:**
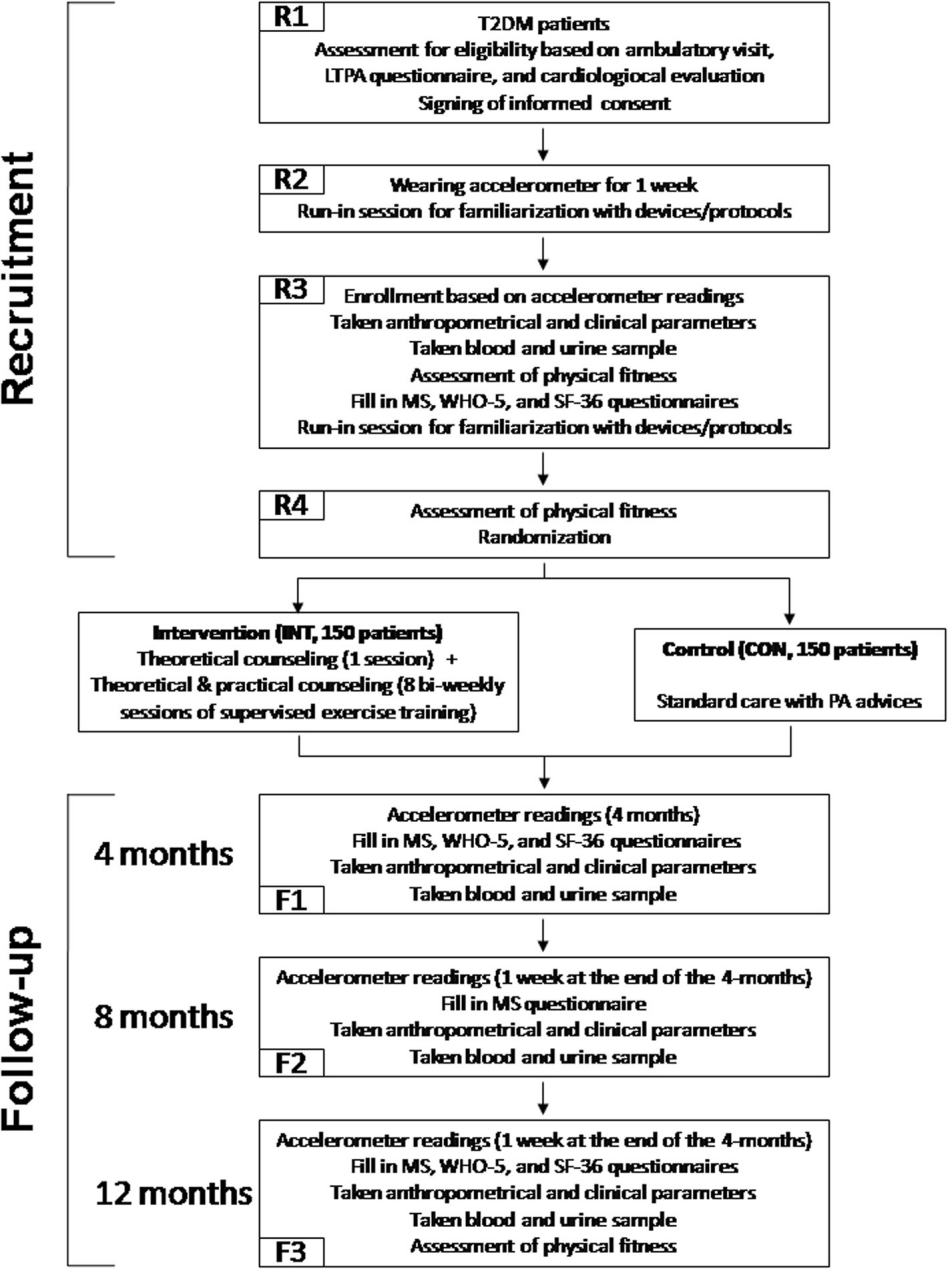
Study flow chart. Sequence of recruitment and follow-up visits during year 1. Follow-up visits F1, F2, and F3 are repeated at years 2 (F4, F5, and F6) and 3 (F7, F8, and F9), except that accelerometer is worn for 1 week at the end of the first 4-month period (F4 and F7). T2DM type 2 diabetes mellitus; LTPA leisure-time physical activity; MS musculoskeletal; WHO World Health Organization; SF Short Form; INT intervention; CON control

The study complies with the Declaration of Helsinki. The research protocol (version #3, 15 February 2012), which follows the SPIRIT guideline (see Additional file 1: Appendix B), has been approved by the Comitato Etico dell’Azienda Ospedaliera Sant’Andrea Prot. n. 212/2012 and written informed consent (see Additional file 1: Appendix C1) is provided by each participant. Participants are not provided with an honorarium. The flow chart of the study is presented in Fig. 1.

### Participants

The main entry criterion is known T2DM (defined by the ADA criteria) [28] of at least 1-year duration. Additional requirements are age 40 to 80 years, body mass index (BMI) 27 to 40 kg/m^2^, sedentary lifestyle (that is, more than 8 hours/day spent in any waking behavior characterized by an energy expenditure ≤1.5 metabolic equivalents (METs) while in a sitting or reclining posture) [29] and physically inactive (that is, insufficient amounts of PA according to current guidelines) [30] for at least 6 months, ability to walk 1.6 km without assistance, and eligibility after cardiologic evaluation.

In order to preserve high internal validity and reduce the risk of adverse events, the criteria presented in Table 1 are used to exclude individuals with conditions that limit or contraindicate PA, affect conduct of the trial, reduce lifespan, and/or affect the safety of intervention.

**Table 1:** Exclusion criteria

Unable or unwilling to give informed consent or communicate with local study staff
Current diagnosis of psychiatric disorder or hospitalization for depression in the past 6 months
Self-reported alcohol or substance abuse within the past 12 months
Self-reported inability to walk 2 blocks
Musculoskeletal disorders or deformities that may interfere with participation in the intervention
History of central nervous dysfunction such as hemiparesis, myelopathies, or cerebral ataxia
Clinical evidence of vestibular dysfunction
Postural hypotension defined as a fall in BP when changing position of >20 mmHg (systole) or >10 mmHg (diastole)
Currently pregnant or nursing
Cancer requiring treatment in the past 5 years, except for cancers that have clearly been cured or in the opinion of the investigator carry an excellent prognosis (for example, stage 1 cervical cancer)
Chronic obstructive pulmonary disease
End-stage liver disease;
Chronic diabetic complications:
• recent major acute cardiovascular event, including heart attack or stroke/transient ischemic attack(s), revascularization procedure, or participation in a cardiac rehabilitation program within the past 3 months • pre-proliferative and proliferative retinopathy
• macroalbuminuria and/or eGFR < 45 ml/min/1.73 m^2^
• severe motor and sensory neuropathy
• diabetic foot with history of ulcer
• Cardiovascular disease at cardiologic examination:
• history of cardiac arrest
• history of pulmonary embolism in the past 6 months
• unstable angina pectoris or angina pectoris at rest
• resting HR <45 beats/min or >100 beats/min
• complex ventricular arrhythmia at rest or with exercise
• uncontrolled atrial fibrillation (HR ≥100 beats/min)
• NYHA Class III or IV congestive heart failure
• acute myocarditis, pericarditis or hypertrophic myocardiopathy
• left bundle branch block or cardiac pacemaker
Conditions not specifically mentioned above at the discretion of the clinical site

Criteria for excluding patients for entry into the IDES-2

*IDES* Italian Diabetes and Exercise Study, *BP* blood pressure, *eGFR* estimated glomerular filtration rate, *HR* heart rate, *NYHA* New York Heart Association

### Investigators

A specific strategy was implemented to train physicians and exercise specialists involved in the IDES and IDES_2 in order to improve efficacy and safety of intervention and patient adherence and to minimize drop-out. The training path dedicated to physicians (diabetologists) was aimed at increasing their awareness of the importance of PA/exercise as a preventive and therapeutic measure in T2DM and other metabolic disorders as well as at instructing them on how to administer a structured exercise counselling and prescribe PA/exercise to subjects suffering from these disease conditions. The training path dedicated to exercise specialists was aimed at instructing professionals with a Bachelor’s degree in exercise science on how to assess physical fitness and PA volume and to supervise exercise sessions, including monitoring of clinical parameters, in individuals with metabolic disorders. At the end of the training program, exercise specialists were certified for assisting these patients in their participation to exercise training programs. All these activities were proposed and managed by the Metabolic Fitness Association and were detailed in the online supplemental material of a previous IDES publication [14]. To minimize drop-out and reduce the attrition bias due to missing data, both physicians and exercise specialists have been specifically instructed on how to promote participant retention in the trial. In particular, investigators have been asked to contact participants at regular intervals, and subjects from both groups are offered access to intervention after the trial. Moreover, investigators have been instructed to keep up-to-date contact information for participants and to collect complete data for both the primary and secondary outcomes, regardless of whether subjects continue to receive the assigned treatment. This strategy was successfully applied to the IDES, in which adherence was high and drop-out was low [14].

### Recruitment

Three-hundred patients are being recruited in three tertiary referral outpatient Diabetes Clinics in Rome beginning in January 2014 (see Additional file 1: Appendix A). All patients attending these clinics are evaluated for eligibility. The recruitment process includes four visits designated as R1, R2, R3, and R4 (Table 2).

**Table 2:** Study schedule

	Study period															
	Recruitment and allocation				Follow-up											
	Recruitment			Allocation	Post-allocation											Close-out
Visit	R1	R2	R3	R4	yr 1	F1	F2	F3	yr 2	F4	F5	F6	yr 3	F7	F8	F9
Time	−4 wks	−3 wks	−2 wks	−1 wk	mo 1	mo 4	mo 8	mo 12	mo 13	mo 16	mo 20	mo 24	mo 25	mo 28	mo 32	mo 36
Enrollment																
Eligibility screen																
Medical history	X															
Clinical examination	X															
LTPA questionnaire	X															
Confirmation of sedentary habits/ physical inactivity			X													
informed consent	X															
Demographics	X															
Cardiologic evaluation	X															
Allocation				X												
Interventions																
CON group																
Physician recommends increasing daily PA				X					X				X			
INT group																
Theoretical session				X					X				X			
Theoretical and practical sessions					X				X				X			
Both: Standard care																
Prescription			X													
Adjustment						X^a^	X^a^	X^a^		X^a^	X^a^	X^a^		X^a^	X^a^	X^a^
Assessments																
Physical fitness																
Run-in familiarization session		X	X													
Assessment				X				X				X				X
Accelerometer (key)																
Delivery of the key (and daily diary)		X		X			X	X		X	X	X		X	X	X
Return of the key (and daily diary)			X			X	X^a^	X^a^		X^a^	X^a^	X^a^		X^a^	X^a^	X^a^
CV risk factors																
Anthropometric data			X			X	X	X		X	X	X		X	X	X
Bioimpedence			X			X	X	X		X	X	X		X	X	X
Blood pressure			X			X	X	X		X	X	X		X	X	X
Blood and urine testing			X			X	X	X		X	X	X		X	X	X
Questionnaires																
MS			X			X		X		X		X		X		X
WHO-5			X			X		X		X		X		X		X
SF-36			X			X		X		X		X		X		X

Overview of the study visits and assessments in the IDES_2

^a^Three to 10 days after the visit

*IDES* Italian Diabetes and Exercise Study, *LTPA* leisure time physical activity, *CV* cardiovascular, *MS* musculoskeletal, *WHO* World Health Organization, *SF* Short Form, *CON* control, *INT* intervention

On R1, patients fulfilling the above criteria are identified based on medical history, clinical examination, and results of the Minnesota LTPA questionnaire. Then, patients are asked to sign a statement of informed consent, are registered in the IDES_2 database available at http://www.metabolicfitness.it/, and undergo a cardiologic examination for cardiovascular evaluation, including a resting ECG and, based on clinical judgment, an echocardiogram and/or an ECG treadmill test.

On R2, in order to objectively verify sedentary habits and physical inactivity, eligible patients are asked to wear an accelerometer (MyWellness Key, Technogym, Gambettola, IT) for 1 week [31, 32] and to report in a daily diary the hours spent wearing the instrument and those spent sleeping and napping. Then, patients attend a run-in session for familiarization with testing devices and protocols for the assessment of physical fitness.

On R3, patients are enrolled after confirming eligibility on the basis of the accelerometer readings. Then, the baseline anthropometrical and clinical parameters and blood and urine samples for biochemical testing are taken, and based on these values, patients receive a standard treatment regimen including nutritional therapy and prescription of pharmacological agents as needed. Subsequently, participants are asked to fill in three questionnaires: a 50-item self-report questionnaire for musculoskeletal (MS) symptoms [33], the World Health Organization (WHO)-5 Well-being Index, a five-item questionnaire for screening of depression [34]; and the Short Form (SF)-36 health survey, with eight scales and two summary measures evaluating physical and mental health-related QoL [35]. Finally, participants perform another run-in familiarization session for physical fitness assessment.

On R4, all participants undergo baseline assessment of physical fitness, are informed about group assignment, receive theoretical counseling or PA advices, according to the study group, and are given an accelerometer to be worn for 4 months, together with a daily diary to report on the hours spent wearing the instrument, sleeping and napping, and performing non-accelerometer recordable PAs.

### Randomization

Patients are randomized 1:1 to an intervention (INT) group (*n* = 150), receiving a theoretical and practical exercise counseling on top of standard care, and a control (CON) group (*n* = 150), receiving only standard care including general physician recommendations for daily PA.

Randomization is stratified by center and, within each center, by age and type of diabetes treatment (non-insulin versus insulin therapy), using a permuted-block randomization software that randomly varies the block size. To ensure allocation concealment, randomization is centralized at the Center for Outcomes Research and Clinical Epidemiology (CORE). After randomization, participants, physicians and exercise specialists are unblinded to group assignment, as blinding is not feasible in exercise-intervention studies.

### Follow-up

Participants from both groups will attend nine follow-up visits, F1 to F9, at 4, 8, 12, 16, 20, 24, 30, and 36 months, respectively (Table 2).

At F1, patients return the key and daily diaries; anthropometrical and clinical parameters and blood and urine samples are taken; and patients are asked to fill in the MS, WHO-5, and SF-36 questionnaires. Three to 5 days later, patients are seen again to discuss accelerometer readings, with reinforcement of exercise counseling (for the INT group) or PA advices (for the CON group), as well as clinical and laboratory data, with eventual adjustment of dietary and pharmacological prescriptions.

At F2, F3, F4, F5, F6, F7 and F8, anthropometrical and clinical parameters and blood and urine samples are taken, and patients are asked to fill in the MS, WHO-5, and SF-36 questionnaires (the latter two only at F3, F4, F6 and F7), and are given an accelerometer (to be worn for 7 days), together with the daily diary. At F4 and F7 only, participants repeat the assessment of physical fitness. Seven to 10 days later, patients are seen again to collect the key and daily diaries and to discuss accelerometer readings, with reinforcement of exercise counseling (for the INT group) or PA advices (for the CON group). As during the F1, results of clinical and laboratory evaluation are discussed, with eventual adjustment of dietary and pharmacological prescriptions.

At F9, patients are given an accelerometer (to be worn for 7 days), together with the daily diary. Seven to 10 days later, patients are seen again to collect the key and daily diaries and to obtain anthropometrical and clinical parameters, blood and urine samples, and measurement of physical fitness at end-of-study. Patients are also asked to fill in the MS, WHO-5, and SF-36 questionnaires for the final evaluation.

### Intervention

The intervention in the INT group consists of aggregated behavioral-change techniques (one individual theoretical exercise counseling session plus eight individual theoretical and practical exercise-counseling sessions) once-a-year for 3 years.

This approach has been designed based on the social cognitive theory and health belief model and uses several behavioral-change techniques [36] (Table 3).

**Table 3:** Characteristics of behavioral intervention

	Theoretical exercise counseling session	Theoretical and practical exercise counseling sessions
Theory of behavior change	Social Cognitive Theory	Social Cognitive Theory
	Health belief model	Health belief model
Behavior change techniques	Provide information on consequences of behavior in general and individual [1, 2]	Provide information on consequences of behavior in general and individual [1, 2]
	Goal setting (behavior) [5]	Goal setting (behavior) [5]
	Goal setting (outcome) [6]	Goal setting (outcome) [6]
	Barrier identification/problem solving [8]	Action planning [7]
	Set graded tasks [9]	Barrier identification/problem solving [8]
	Prompt review of behavioral goals [10]	Prompt review of behavioral goals [10]
	Prompting generalization of a target behavior [15]	Prompt review of outcome goals [11]
	Prompt self-monitoring of behavior[16]	Prompting generalization of a target behavior [15]
	Provide information on where and when to perform the behavior [20]	Prompt self-monitoring of behavior [16]
	Motivational interviewing [37]	Prompt self-monitoring of behavioral outcome [17]
		Provide information on where and when to perform the behavior [20]
		Provide instruction on how to perform the behavior [21]
		Model/demonstrate the behavior [22]
		Time management [38]
Delivery of the intervention	Individual face-to-face session	Individual face-to-face sessions
	Physician interventionist	Exercise specialist interventionist
	One 30-min session once a year for 3 years	Eight 75-min sessions once a year for 3 years

Theories of behavior change, behavior change techniques, and mode of delivery of intervention in the IDES_2

Numbers in the square brackets correspond with the code assigned to each behavior change technique described in ref #36

#### Theoretical counseling sessions

The theoretical, individual, face-to-face counseling session has been previously validated [27] and tested successfully in clinical settings [27, 37]. In this session, which is held in each Diabetes Clinic by a trained diabetologist and lasts 30 min, the physician follows the checklist presented in Table 4 [27].

**Table 4:** Checklist of the theoretical counseling session

#	Item	Content
1	Motivation	The benefits of PA/exercise are described as reported by the scientific literature for diabetic patients, stressing those appealing most to the individual patient. Efforts are designed to convince the patient that regular PA is the pre-eminent cure for T2DM as well as to understand what positive expectations the individual patients held from this change in behavior. The importance of reducing SED-time is also stressed.
2	Self-efficacy	Self-efficacy is promoted by patient collaboration in designing an individualized program of PA, based on age and physical state and setting realistic personal goals.
3	Pleasure	Based on the patient’s previous experience of PA/exercise, a choice of several interchangeable indoor and outdoor PAs is proposed to identify those that are more appealing.
4	Support	The supportive presence of a partner/family member/group of peers is preferred, and we offer eight structured indoor PA/exercise sessions in the gym of the Metabolic Fitness Association.
5	Comprehension	Feedback from the patients is elicited to check if they really understand the valuable advantages of the behavioral change. After the exercise program is established, the patient is questioned to establish whether there is a really positive attitude toward the behavioral change. Care is taken to recognize uncertainties and identify perceived impediments to PA.
6	Lack of impediments	Potential obstacles to regular PA/exercise are identified. Instead of simply suggesting a solution, patients are invited to solve the problem and their proposals are supplemented with advice on time management strategies.
7	Diary	The patient are asked to record daily the type and time of PA they perform. On the subsequent visits (every 4 months), the diary is used to record the amount of PA, to encourage patients’ self-efficacy, to increase the time or frequency of PA and to overcome practical problems related to PA/exercise.

*PA* physical activity, *T2DM* type 2 diabetes mellitus, *SED*-*time* sedentary time

### Theoretical and practical counseling sessions

The practical counseling intervention program consists of eight distinct but interrelated modules that cover topics such as diabetes, free living PA and exercise and the psychology of behavior change. The exercise sessions (75 min each), held in three specialized gym facilities (see Additional file 1: Appendix A) and supervised by a certified exercise specialist, are geared to expand and put into practice the knowledge acquired during the theoretical counseling session. Each exercise session is composed of 30 min of aerobic exercise followed by 30 min of resistance exercise, plus additional 15 minutes for warm-up and cool-down (including stretching).

In these sessions, the exercise specialist improves the patient’s knowledge of the effects of exercise on patient’s own health, including both advantages and disadvantages; conditions under which exercise should not be done; difference between habitual and occasional exercise; essential parameters of wellness such as blood pressure (BP), heart rate (HR), and blood glucose. In addition, health instructions are given to identify the difference between aerobic and resistance exercise; evaluate the intensity of exercise; perform warm-up and stretching safely; monitor blood glucose correctly; and correct imbalances during and after the exercise session. A detailed list of the actions performed by the exercise specialist during the theoretical and practical counseling sessions is presented in Table 5.

**Table 5:** List of the actions performed by the exercise specialist during the theoretical and practical counseling sessions

#	Action
1	Establishes clinical condition and physical fitness and identifies the adequate exercise protocol (using a homemade exercise algorithm) on the basis of previous evaluations.
2	Measures BP, HR, and glucose level before and after each exercise session and provides feedback to the subject on the effect of the specific exercise adopted.
3	Instructs the patient to perform these measurements, indicates the range of glycemic values allowing or contraindicating exercise, and suggests to start with resistance exercise when glycemia is on the low side of the range, especially for those treated with insulin or secretagogues.
4	Reviews with the patient the structure of the exercise session (warm-up, exercise, and cool-down phases), instructs on how to perform a safe warm-up and cool down, providing practical examples for the different exercise types, and stressing the importance of the gradualism of the increase and decrease of the intensity for the warm up and cool down, respectively.
5	Explains the difference between aerobic and resistance exercise, proving practical examples of the different exercise forms (endurance exercise machines, resistance exercise devices, free body exercises).
6	For aerobic exercise training, illustrates the correct exercise progression and control and, throughout the counseling sessions, encourages the patients to progressively increase the exercise intensity, instructing on how to identify and control light, moderate and vigorous intensity on the basis of breathing frequency (talk test), rate of perceived exercise, and HR. As an example, exercise intensity is explained on the basis of the ability to perform a conversation as follows: o light intensity: “allows to respond to conversation without problems.” o moderate intensity: “allows to carry on a conversation but with some difficulties.” o vigorous intensity: “ the speech is limited to short phrases.”
7	Especially for unsupervised PA/exercise and for patients with complications, advises the patients to work at low-to-moderate intensity, as it is the one allowing to reduce the risk of adverse events while providing substantial benefits.
8	With regard to the volume of aerobic exercise, gives examples on how to comply with the exercise recommendations on the minimum amount of PA/exercise providing health benefits, and stressing that adding more PA/exercise will results in additional benefits.
9	Describes the correct way of increasing training volume, augmenting first duration and then intensity, especially in patients with complications.
10	Explains the difference between weight bearing and non-weight-bearing exercise and the relevance for the subjects with complications, providing practical examples.
11	For resistance exercise training, provides examples on the exercise taxing the major muscle groups (eight to 10 exercises), explaining (and checking the learning of) the correct exercise technique, and illustrates a typical sequence of resistance exercises, alternating opposing muscle groups and/or upper and lower body exercises.
12	Explains the concept that a higher repetitions number corresponds to a light weight to be lifted (therefore lighter intensity) and vice versa, and teaches the correct breathing pattern during the different resistance exercises adopted, reminding that breath holding (Valsalva maneuver) should be avoided.
13	Instructs to perform multiples sets (for example, two to three sets) with adequate recovery between them (for example, 2 min), depending on the intensity adopted (less repetitions per set usually equal longer recovery time).
14	Identifies indicated and contraindicated resistance exercises on the basis of the specific patient’s complications.
15	Provides examples on how to substitute the typical exercises executed in the gym setting with other forms of PA/exercise to be performed outside the gym, reminding patients that new forms of unsupervised PA/exercise should be adopted after consulting the exercise specialist.
16	Helps the patient to organize a typical working day and weekend in order to find time and space for performing any type of PA (home, commuting, occupational, and LT), reduce the SED-time, and remove potential obstacles.
17	Assists the patient in setting behavior and outcome goals as well as in the choice of the indoor and outdoor PAs that are the most appealing and feasible.

*BP* blood pressure, *HR* heart rate, *PA* physical activity

### Standard care

Participants from the CON group receive general physician recommendations for increasing the amount of daily PA and decreasing the SED-time.

All patients receive a treatment regimen aimed at achieving optimal glycemic, lipid, BP, and body weight targets, as established by current guidelines [28]. This program eventually includes glucose-, lipid- and BP-lowering agents as needed and, when indicated, anti-platelet drugs, plus a dietary prescription, which is preceded by a preliminary nutritional evaluation with assessment of BMI and the individual patient requirements and preferences. The diet contains 55 % calories from complex carbohydrates, 30 % from fat, and 15 % from protein. Since all patients are overweight or obese, they receive a low-calorie diet (negative balance of 500 kcal/day, but not less than 1500 kcal in males and not less than 1200 kcal in females), calculated by adding the estimated energy expenditure from routine and voluntary PA to the basal metabolism estimated using the gas exchange analyzer FitMate (Cosmed, Rome, Italy). PA is calculated by multiplying the MET scores (one MET expresses an oxygen consumption, taken by convention to be 3.5 ml) of the various activities by the weekly hours spent in each of them, as reported in the Minnesota LTPA questionnaire, and expressed as METs ∙ h^−1^ ∙ week^−1^.

### Outcome measures

The primary objective of IDES-2 is to evaluate the incremental impact of intervention on the promotion and maintenance of a physically active lifestyle as well as on the reduction in SED-time.

Secondary objectives include testing the efficacy of the intervention on physical fitness, modifiable cardiovascular risk factors, musculoskeletal disturbances, well-being/depression, and health related QoL.

As physicians and exercise specialists are aware of group assignment, outcome assessment is also unblinded, except for biochemical testing at the central laboratory.

### Measurements

#### Assessment of PA

At screening, the volume of PA is assessed retrospectively using the Minnesota LTPA questionnaire [38]. At baseline, follow-up, and end-of-study, each participant is outfitted with a uniaxial piezoelectric accelerometer MyWellness Key (Technogym, Gambettola, IT) [31]. The advantages of using the MWK accelerometer relies on the fact that it offers the possibilities of storing 30 days of continuous movement detection while exercise intensity detection is aligned with other laboratory validations [32, 39]. In addition, it was recently shown to measure PA volume accurately and to acceptably discriminate between low- and moderate-intensity PA in individuals with T2DM [40]. The device is attached at the waistband in midline of the right anterior hip and detects the minutes spent at light, moderate and vigorous intensities and the total volume of PA [32]. Data are collected and downloaded on a dedicated web-portal. Each participant wears the device for 4 months at baseline and for 7 consecutive days every 4 months thereafter. Upon waking, participants attach the device (immediately after bathing or showering) and wear it all day (except if swimming) up to bedtime, for at least 10 h/day. Patients are also asked to report on a daily diary the hours spent wearing the instrument, sleeping and napping, and performing nonaccelerometer recordable PAs such as swimming, cycling, skiing, etc.

The time the patient is awake and is not wearing the accelerometer, is assumed to be spent in sedentary activities (for example, taking a shower or getting dressed), unless spent in PAs that cannot be performed while wearing the accelerometer (for example, swimming). SED-time is then calculated by adding this time to that recorded by the accelerometer with readings <100 counts/min, a threshold that corresponds with sitting, reclining, or lying down, that is, to <1.5 METS [41].

Matthews’ cut-points are used to identify the time spent in light intensity (100 to 1951 counts/min corresponding to 1.5 to 2.9 METs) [42], whereas Freedson’s cut-points are used to determine the time spent in PA of moderate intensity (1952 to 5724 counts/min corresponding to 3 to 5.9 METs) and vigorous intensity (≥5725 counts/min corresponding to ≥6 METs) [43]. The time spent in non-accelerometer-recordable PAs, as reported on the daily diary, is added to that recorded by the accelerometer, according to the intensity of each activity [44]. Moderate-intensity PA is combined with vigorous-intensity PA into the MVPAs, as participants spend little time in vigorous-intensity PA.

#### Assessment of physical fitness

Parameters of physical fitness, that is, cardiorespiratory fitness, strength and flexibility, are evaluated at baseline, 12 months, 24 months, and end-of-study. The tests are preceded by two consecutive run-in sessions to allow the patients to become familiar with the testing devices and protocols.

Cardiorespiratory fitness is assessed by a maximal treadmill exercise test using a Balke protocol [45]. The treadmill speed is set at 88 m/min and the grade is 0 % during the first minute, 2 % during the second minute, and is then increased every minute by 1 %; after 25 minutes, the treadmill grade is kept constant, whereas the speed is increased by 5.4 m/min every minute. Patients are instructed not to hold the treadmill rails and are verbally encouraged to reach volitional exhaustion, as reported by the patient or by the physician for medical reasons. The time to exhaustion is used to estimate the maximal oxygen uptake, given the high correlation between these two parameters [46].

For strength assessment, isometric muscle strength is measured by means of a strain gauge tensiometer (Digimax, Mechatronic GmbH, Germany), as previously described [47]. Isometric tests have been chosen since they are reproducible [48, 49], do not require complex execution technique, and can therefore be easily performed by both trained and untrained subjects. Lower limb muscle strength is assessed by maximal voluntary contractions (MVCs) performed at a costumed leg extension machine (Technogym). The angle at the knee and the hip is fixed at 90°. Upper body muscle strength is assessed by MVCs performed at a shoulder press (Technogym) along the sagittal plane, with a 90° and 45° angle at the elbow and between the upper arm and the trunk, respectively. These exercise modalities have been chosen in order to test muscle chains involved in the fundamental activities of daily living. In both tests, subjects are asked to exert their maximal force with both limbs simultaneously and to maintain it for at least 2–3 s before relaxing. For each exercise, three MVC are performed, with a 3-min rest interval between contractions. Participants are asked to perform additional contractions if the MVC of their last attempt exceeds the previous by at least 5 %. During the MVCs, verbal encouragement is provided, always by the same investigator for all subjects.

For hip and trunk flexibility assessment, a standard bending test is executed [27]. Standing on a step with legs fully extended, patients are asked to bend the torso forward to try to touch the ground with their fingertips. The test is performed three times and the distance between the finger and the ground is measured by the exercise specialist at the third attempt.

#### Assessment of modifiable cardiovascular risk factors

The following modifiable cardiovascular risk factors are being evaluated at baseline, end-of-study, and every 4 months in-between: HbA_1c;_ fasting plasma glucose (FPG); serum insulin; C-peptide; BMI; waist circumference; body composition; BP; triglycerides; total, LDL and HDL cholesterol; high sensitivity-C-reactive protein (hs-CRP); aspartate aminotransferase (AST); alanine aminotransferase (ALT); γ-glutamyl-transpeptidase (γ-GT); creatine kinase (CK); complete blood count; uric acid; blood urea nitrogen (BUN); serum creatinine; urinalysis; and albumin/creatinine ratio on first-voided urine samples.

Body weight and height are measured using a scale and stadiometer, and BMI is then calculated as weight (kg)/height (m^2^), whereas waist circumference is taken at the umbilicus. Body composition is evaluated by assessing the fat mass and fat-free mass by the use of a body fat monitor (Tanita BF664, Vernon Hills, IL, USA). BP is recorded with a sphygmomanometer after a 5-minute rest while the patient remains seated with the arm at the heart level.

Biochemical tests are centralized at the Laboratory of Clinical Chemistry of Sant’Andrea Hospital, an accredited and ISO9001 certified structure, using standard analytical techniques (Table 6). The Homeostasis Model Assessment-Insulin Resistance (HOMA-IR) index is calculated from the FPG and insulin levels [50]. The glomerular filtration rate (GFR) is estimated from the serum creatinine by the use of the Chronic Kidney Disease Epidemiology Collaboration (CKD-EPI) equation [51]. Global and fatal CHD 10-year risk scores are calculated using the United Kingdom Prospective Diabetes Study (UKPDS) risk engine [52].

**Table 6:** Laboratory tests

Analyte	Method	Manufacturer
HbA_1c_	HPLC (Adams TMA1C HA-8160)	Menarini Diagnostics, Florence, Italy
FPG	VITROS 5,1 FS Chemistry System	Ortho Clinical Diagnostics Inc, Raritan, NJ, USA
Insulin	Chemiluminiscent immunometric assays (Immulite 2000 Thes)	Diagnostic Products Corporation, Los Angeles, CA, USA
C-peptide	Chemiluminiscent immunometric assays (Immulite 2000 Thes)	Diagnostic Products Corporation, Los Angeles, CA, USA
Triglycerides	VITROS 5,1 FS Chemistry System	Ortho Clinical Diagnostics Inc, Raritan, NJ, USA
Total cholesterol	VITROS 5,1 FS Chemistry System	Ortho Clinical Diagnostics Inc, Raritan, NJ, USA
LDL cholesterol	VITROS 5,1 FS Chemistry System	Ortho Clinical Diagnostics Inc, Raritan, NJ, USA
HDL cholesterol	VITROS 5,1 FS Chemistry System	Ortho Clinical Diagnostics Inc, Raritan, NJ, USA
hs-CRP	VITROS 5,1 FS Chemistry System	Ortho Clinical Diagnostics Inc, Raritan, NJ, USA
AST	VITROS 5,1 FS Chemistry System	Ortho Clinical Diagnostics Inc, Raritan, NJ, USA
ALT	VITROS 5,1 FS Chemistry System	Ortho Clinical Diagnostics Inc, Raritan, NJ, USA
γ-GT	VITROS 5,1 FS Chemistry System	Ortho Clinical Diagnostics Inc, Raritan, NJ, USA
CK	VITROS 5,1 FS Chemistry System	Ortho Clinical Diagnostics Inc, Raritan, NJ, USA
Complete blood count	VITROS 5,1 FS Chemistry System	Ortho Clinical Diagnostics Inc, Raritan, NJ, USA
Uric acid	VITROS 5,1 FS Chemistry System	Ortho Clinical Diagnostics Inc, Raritan, NJ, USA
BUN	VITROS 5,1 FS Chemistry System	Ortho Clinical Diagnostics Inc, Raritan, NJ, USA
Serum creatinine	VITROS 5,1 FS Chemistry System	Ortho Clinical Diagnostics Inc, Raritan, NJ, USA
Urinary albumin	mAlb VITROS	Ortho Clinical Diagnostics Inc, Raritan, NJ, USA
Urinary creatinine	VITROS 5,1 FS Chemistry System	Ortho Clinical Diagnostics Inc, Raritan, NJ, USA

Biochemical measurements in the IDES-2

*IDES* Italian Diabetes and Exercise Study, *HbA*_*1c*_ hemoglobin A_1c_, *HPLC* high-performance liquid chromatography, *FPG* fasting plasma glucose, *hs*-*CRP* high sensitivity-C-reactive protein, *AST* aspartate aminotransferase, *ALT* alanine aminotransferase, *γ*-*GT* γ-glutamyl-transpeptidase, *CK* creatine kinase, *BUN* blood urea nitrogen

#### Assessment of musculoskeletal disturbances

Musculoskeletal symptoms are evaluated by the use of a 50-item self-report questionnaire (see Additional file 1: Appendix C2) [33], which investigates shoulder, arm, elbow, wrist, hand, spine, hip, knee, ankle, and foot problems.

#### Assessment of well-being/depression

Screening for depression is performed by asking participants to fill in the WHO-5 Well-being Index, a five-item questionnaire (see Additional file 1: Appendix C3) [34], which was found to be suitable for use in outpatients with type 1 and T2DM [53].

#### Assessment of health-related QoL

Health-related QoL is evaluated using the Italian version [54] of the SF-36 health survey (ISF-36) (see Additional file 1: Appendix C4) [35], which yields four physical and four mental health component scores and has been previously validated in subjects with T2DM [55, 56]. The physical and mental component summary measures (PCS and MCS, respectively) are normalized to a population mean of 50 and SD of 10.

### Adverse events

Adverse events are reported at intermediate visits and, for INT subjects, also at supervised sessions, by completing a standard form.

### Dissemination policy

Trial results will be communicated to participants via the website of the Metabolic Fitness Association (http://www.metabolicfitness.it/) and by organizing a dedicated event. Results will by also communicated to the scientific via publication and to the public via press release. The final report will follow the main CONSORT 2010 guideline and will be extended as necessary for a non-pharmacological intervention.

After publication of the results, public access to the full protocol, participant-level dataset, and statistical code will be eventually granted upon request.

### Data collection, storage and security

Data collected via web into the IDES_2 database and downloaded from the accelerometers are saved to a password-protected server in the Metabolic Fitness Association. These data are accessed only by members of the research team. Once all data have been uploaded to the server, they will be securely deleted from the recording devices. Patient questionnaire data will be anonymized and stored in locked filing cabinets in the Metabolic Fitness Association.

### Statistical analysis

Sample size calculation is based on preliminary accelerometer data showing that daily PA in sedentary, physically inactive T2DM patients is 24.2 ± 9.4 METs ∙ h^−1^ ∙ wk^−1^. To observe a 15 % increase in daily PA with a statistical power of 90 % (α = 0.05) by unpaired t-test, 142 patients per arm are needed (284 total). A sample size of 300 patients will allow supporting a 5 % dropout rate, as detected in the EXE group from the IDES [14].

The χ^2^ test for categorical variables and the Student’s t test or the corresponding nonparametric Mann–Whitney test for continuous variables will be utilized to compare patients’ characteristics at baseline.

The intent-to-treat analysis for primary and secondary endpoints will be applied to all randomized patients with a baseline and at least one post-baseline value. The efficacy of the intervention, as compared with standard care, on the primary and secondary endpoints will be assessed by a repeated measures analysis of covariance (ANCOVA - using PROC MIXED in SAS software) of change from baseline to the end-of-study. In case of differences between groups in baseline values, we will consider a baseline adjustment to improve power. The differences between end-of-study and baseline QoL scores and summary measures between the two groups will be compared using the Mann–Whitney U-test, while changes in QoL parameters within each group (end-of-study versus baseline) will be tested using the Wilcoxon test.

To account for change in medication throughout the study period, which might affect performance with respect to cardiovascular risk factors, we will perform both multiple regression and sensitivity analyses. In the regression models, the dependent variable will be represented by baseline to end-of-study changes. Treatment at baseline and treatment initiation during the study will be included in the model as dichotomous variables (yes versus no), whereas drug dosage will be not taken into consideration. Sensitivity analysis will be conducted by comparing study arms after exclusion of the patients who modified the treatment.

In order to treat attrition, we will assume that data are missing at random, use adjustment methods for missing data, and conduct a sensitivity analysis to assess the robustness of inferences about treatment effects to various missing-data assumptions [57]. In particular, repeated measures models with an autoregressive correlation type matrix will be applied in order to account for both missingness at random and potential correlation within subjects. These methods are more efficient in that they allow evaluating all subjects, even those with incomplete data [58, 59]. Moreover, to guarantee replicability and avoid outcome selective reporting, a fully specified statistical analysis plan will be written before unmasking.

Statistical analyses will be performed by at the CORE using SAS software release 9.3 (Cary, NC, USA).

## Discussion

Meta-analyses of small-sized studies showed that structured aerobic exercise is effective in improving cardio-respiratory fitness [60] as well as glycemic control and other modifiable cardiovascular risk factors [61, 62] in subjects with T2DM. These data suggested that additional benefits, beyond those of the PA itself, may be provided by exercise (that is, by planned or structured PA that involves repetitive bodily movements performed to improve or maintain one or more of the components of physical fitness, such as cardiorespiratory and muscular fitness) [8]. Based on these early studies [60–62], as well as on subsequent RCTs [11, 12, 14–16] and a large meta-analysis [13], PA/exercise has become an integral part of T2DM management [7–10].

However, implementation of PA/exercise in routine clinical practice for adults with T2DM is hindered by several impediments [21]. Compliance to PA/exercise recommendations is usually poor in sedentary and physically inactive patients such as those suffering from metabolic disorders, since sustained changes in lifestyle may require much time and effort. In fact, both RCTs and observational studies showed that health benefits from PA/exercise are volume-dependent, since they start beyond a certain threshold, increase almost linearly thereafter, and finally plateau as large volumes are achieved [14, 26, 63]. However, in the long-term, beneficial effects might be obtained also with smaller volumes of daily PA. Previous studies showed that an increase of 1 MET was associated with approximately an 18 % reduced risk of death [64, 65] and an increase of 1,000 steps resulted in a reduction of postprandial blood glucose by 1.6 mmol/L over a period of 2 years [66]. In addition, in the European Prospective Investigation into Cancer and Nutrition Study (EPIC), the greatest reductions in mortality risk were observed between the two lowest activity groups across levels of total and abdominal adiposity, suggesting that efforts to encourage even small increases in activity in sedentary/inactive individuals may be beneficial to public health [67]. Finally, increasing overall PA may have beneficial effects on disease progression and cardiovascular risk in patients with T2DM also by decreasing the amount of SED-time [19, 20].

Thus, behavioral strategies favoring of an increase of PA/exercise as well as a reduction in SED-time may be successful in these individuals, as shown in trials for T2DM prevention [22, 23] and treatment [24]. To this end, counseling interventions focused exclusively on PA were found to be more effective in promoting PA than those targeting multiple behaviors [68] and diabetes self-management education programs were shown to provide clinically meaningful improvements in glycemic control when combined with ≥11 contact hours with delivery personnel [69]. Unfortunately, acceptable, feasible, and validated behavioral interventions are lacking. Available studies are heterogeneous in that they differ on the theory of behavior change used to underpin them (for example, the transtheoretical model, social cognitive theory, or precede/proceed model); the behavior change techniques used to promote change (for example, goal setting, use of follow-up prompts, or provision of feedback on performance); and the modalities of intervention delivery (for example, frequency and duration of contact and one-to-one versus group delivery) [25]. In other circumstances, behavioral interventions have omitted details on these items and also on how intervention was operationalized and evaluated, thus limiting its efficacy and replication outside the research setting [25]. Finally, available studies generally involved small samples for short periods, and in most of them, changes in PA were not objectively measured. PA and SED-time variables in these studies have typically been derived from self-report measures, generally a 1-week recall. In addition to the imprecision associated with such measures, it is also difficult to accurately capture the total sedentary behavior or light intensity PA by questionnaire [70]. Light-intensity PA, which includes activities such as washing dishes, ironing, and other routine domestic or occupational tasks, is the predominant determinant of variability in total daily energy expenditure [71].

Barriers that are outside the patient’s own control include lack of specific knowledge on the part of both physicians and exercise trainers and lack of dedicated facilities. In particular, these is a lack of evidence-based training programs to equip healthcare professionals with a range of intervention-specific competencies (knowledge and skills) and confidence to support adults with T2DM to become more physically active in the long-term. A systematic review and meta-analysis of 17 RCTs reported use of treatment fidelity measures in a majority of studies in terms of study design (for example, consistent duration and frequency of sessions across trial arms) and monitoring to improve receipt of intervention components and enactment of intervention–related knowledge and skills by patients. In contrast, treatment fidelity strategies to monitor and improve provider training and delivery of treatment in practice were frequently omitted (for example, use of standardized training materials, observation of intervention implementation during training with intervention providers and recipients) [72]. Training of intervention providers and treatment fidelity assessment are important for ensuring reproducibility of intervention-related skills in clinical practice and increasing the likelihood of implementation in routine clinical care [73, 74].

The IDES_2 is an RCT testing the incremental impact of a behavioral intervention strategy for the promotion and long-term maintenance of free-living PA/exercise as well as for the reduction in SED-time in subjects with T2DM. This strategy is based on solid theoretical grounds and uses several behavior change techniques, two factors which were found to improve effectiveness of behavioral intervention [72]. In addition, physicians and exercise specialists have been trained to administer a structured exercise counselling and prescribe PA/exercise to subjects suffering from metabolic disorders and to supervise exercise sessions, including monitoring of clinical parameters, for these individuals, respectively. Moreover, the large sample size, the long study duration, and the objective measurement of PA using an accelerometer which allows monitoring for long periods of time will provide statistically significant and scientifically robust data on the efficacy of this intervention. Finally, implementation of this program in routine clinical practice is certainly more feasible than that of life-long supervised training programs, the application of which is hampered by the high costs for both health services and patients.

Based on these considerations, we expect that such behavioral intervention would offer a feasible procedure for the promotion and long-term maintenance of free-living PA/exercise as well as the reduction in SED-time and thus meet the call for a change of paradigm to move beyond the limited clinical focus by including theoretically population-based and "real-life" approaches for the management of T2DM.

With regard to possible risks of bias, we anticipate that, according to the Cochrane Collaboration [75], the risk of selection bias is low, due to the use of adequate methods for sequence generation and allocation sequence concealment, although patients who agree to participate in a lifestyle intervention program might be different from those who refuse. Moreover, though blinding of participants and investigators is impossible in exercise intervention studies, the risks of performance, detection, and attrition bias are also limited for several reasons. Regarding performance, despite the fact that independently of intervention, participants might be influenced by knowledge of group assignment in adopting and maintaining a physically active lifestyle, the possibility that unblinding to group assignment may result in an overestimation or an underestimation of intervention effects is low. On the one hand, subjects from the control group of the IDES performed a considerable amount of PA [14] and, though PA measurements are known to affect physical activity behavior [76], this would impact on PA volume of both groups. On the other hand, investigators are asked not to provide subjects from the control group with PA advice other than general recommendations for increasing the amount of daily PA (and decreasing the SED-time), and intensification of pharmacological treatment to compensate for lower PA in these individuals will be accounted for in the statistical analysis. Regarding detection, because the primary outcome and some secondary outcomes (physical fitness and cardiovascular risk factors) are objective, the risk of bias is low on both the participant’s and investigator’s side, although study subjects might be influenced by knowledge of group assignment when filling in questionnaires for musculoskeletal disturbances, well-being/depression, and health-related QoL. Regarding attrition, in the IDES, knowledge of group assignment did not affect remaining in the trial for the entire study period as participants from the control group dropped out at a rate and for reasons similar to those of the exercise group [14]. Moreover, as reported above, the risk of attrition bias due to missing data has been adequately prevented and will be treated according to a recent National Research Council report on the topic [57].

In conclusion, this trial tests the hypothesis that a theoretical and practical behavioral intervention strategy is more effective than standard care for the promotion and long-term maintenance of free-living PA/exercise as well as for the reduction in SED-time, while reducing modifiable cardiovascular risk factors and improving MS symptoms, well-being, and health-related QoL in T2DM patients.

### Trial status

Recruitment was ongoing at the time of manuscript submission.

## Additional file

Additional file 1:
**See Appendix A for the complete list of the IDES_2 Investigators; Appendix B for the SPIRIT 2013 Checklist; Appendix C for Forms and Questionnaires; and Appendix D for World Health Organization Trial Registration Data Set.** (DOC 252 kb)

## Abbreviations

ACSM: American College of Sports Medicine
ADA: American Diabetes Association
ALT: alanine aminotransferase
AST: aspartate aminotransferase
BMI: body mass index
BP: blood pressure
BUN: blood urea nitrogen
CHD: coronary heart disease
CK: creatine kinase
CKD-EPI: Chronic Kidney Disease Epidemiology Collaboration
CON: control
DARE: Diabetes Aerobic and Resistance Exercise
EPIC: European Prospective Investigation into Cancer and Nutrition Study
FPG: fasting plasma glucose
GFR: glomerular filtration rate
HART-D: Health Benefits of Aerobic and Resistance Training in Individuals with Type 2 Diabetes
HbA1c: hemoglobin A1c
HOMA-IR: Homeostasis Model Assessment-Insulin Resistance
HR: heart rate
hs-CRP: high sensitivity-C-reactive protein
IDES: Italian Diabetes and Exercise Study
INT: intervention
LT: leisure time
METs: metabolic equivalents
MCS: mental component summary
MS: musculoskeletal
MVC: maximal voluntary contraction
MVPA: moderate-to-vigorous PA
NHANES: National Health and Nutrition Examination Survey
PA: physical activity
PCS: physical component summary
QoL: quality of life
RCT: randomized controlled trial
SED-time: sedentary time
SF: short form
T2DM: type 2 diabetes mellitus
UKPDS: United Kingdom Prospective Diabetes Study
WHO: World Health Organization
γ-GT: γ-glutamyl-transpeptidase
