# Supplementary material for: The Italian Diabetes and Exercise Study 2 (IDES-2): a long-term behavioral intervention for adoption and maintenance of a physically active lifestyle
Source: Trials. 2015 Dec 11;16:569. doi: 10.1186/s13063-015-1088-0 (PMC4676117; doi:10.1186/s13063-015-1088-0)
Supplement: Additional file 1: — See Appendix A for the complete list of the IDES_2 Investigators; Appendix B for the SPIRIT 2013 Checklist; Appendix C for Forms and Questionnaires; and Appendix D for World Health Organization Trial Registration Data Set. (DOC 252 kb) [file 13063_2015_1088_MOESM1_ESM.doc]

**Appendix A: List of participants**

**Diabetes Clinics**

1. Diabetes Unit, Sant’Andrea Hospital, Rome, Italy: Giuseppe Pugliese, Maria Cristina Ribaudo, Laura Salvi, Martina Vitale, Lucilla Bollanti, Francesco G. Conti (Coordinating Center).
2. Diabetes Unit, Fatebenefratelli San Pietro Hospital, Rome, Italy: Nicolina Di Biase, Filomena La Saracina.
3. Diabetes Unit, Health District, Monterotondo, Rome, Italy: Stefano Balducci, Mario Ranuzzi, Jonida Haxhi, Valeria D’Errico.

**Metabolic Fitness Centers**

1. Department of Human Movement and Sport Sciences, ‘‘Foro Italico’’ University, Rome, Italy: Massimo Sacchetti, Giorgio Orlando.
2. Metabolic Fitness Association, Monterotondo, Rome, Italy: Gianluca Balducci, Enza Spinelli.
3. Center for the Study of Metabolism, Rome, Italy: Luca Milo, Roberto Milo.

**Central laboratory**

Laboratory of Clinical Chemistry, Sant’Andrea Hospital, Rome, Italy: Patrizia Cardelli, Stefano Cavallo.

**Data management team**

1. Diabetes Unit, Sant’Andrea Hospital, Rome, Italy: Graziela Rangel (data control for completeness and plausibility).
2. Department of Human Movement and Sport Sciences, ‘‘Foro Italico’’ University, Rome, Italy: Massimo Sacchetti, Giorgio Orlando; and School of Science, University of Greenwich, London, UK: Silvano Zanuso (calculation of PA and SED-time).
3. Center for Outcomes Research and Clinical Epidemiology (CORE), Pescara, Italy: Antonio Nicolucci, Giuseppe Lucisano (centralized randomization, data analysis).

**Steering Committee**

Giuseppe Pugliese, Stefano Balducci, Massimo Sacchetti, Silvano Zanuso, Patrizia Cardelli, Antonio Nicolucci.

**Appendix B: SPIRIT 2013 Checklist: Recommended items to address in a clinical trial protocol and related documents***


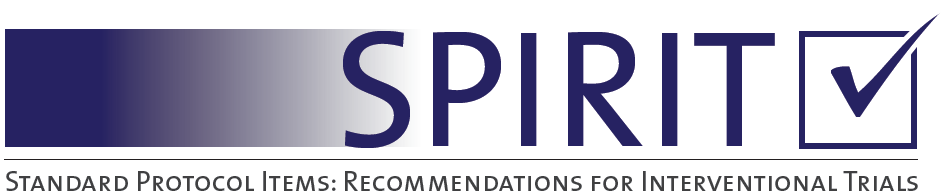


| Section/item | Item No | Description | Addressed on page number |
| --- | --- | --- | --- |
| **Administrative information** | | |  |
| Title | 1 | Descriptive title identifying the study design, population, interventions, and, if applicable, trial acronym | 1 |
| Trial registration | 2a | Trial identifier and registry name. If not yet registered, name of intended registry | 3 |
|  | 2b | All items from the World Health Organization Trial Registration Data Set | Appendix D |
| Protocol version | 3 | Date and version identifier | 7 |
| Funding | 4 | Sources and types of financial, material, and other support | 23 |
| Roles and responsibilities | 5a | Names, affiliations, and roles of protocol contributors | 1, 22-23 |
|  | 5b | Name and contact information for the trial sponsor | 23 |
|  | 5c | Role of study sponsor and funders, if any, in study design; collection, management, analysis, and interpretation of data; writing of the report; and the decision to submit the report for publication, including whether they will have ultimate authority over any of these activities | 23 |
|  | 5d | Composition, roles, and responsibilities of the coordinating centre, steering committee, endpoint adjudication committee, data management team, and other individuals or groups overseeing the trial, if applicable (see Item 21a for data monitoring committee) | Appendix B |
| Introduction |  |  |  |
| Background and rationale | 6a | Description of research question and justification for undertaking the trial, including summary of relevant studies (published and unpublished) examining benefits and harms for each intervention | 4-5 |
|  | 6b | Explanation for choice of comparators | 6 |
| Objectives | 7 | Specific objectives or hypotheses | 5-6 |
| Trial design | 8 | Description of trial design including type of trial (eg, parallel group, crossover, factorial, single group), allocation ratio, and framework (eg, superiority, equivalence, noninferiority, exploratory) | 5 |
| Methods: Participants, interventions, and outcomes | | |  |
| Study setting | 9 | Description of study settings (eg, community clinic, academic hospital) and list of countries where data will be collected. Reference to where list of study sites can be obtained | 8, 11, Appendix B |
| Eligibility criteria | 10 | Inclusion and exclusion criteria for participants. If applicable, eligibility criteria for study centres and individuals who will perform the interventions (eg, surgeons, psychotherapists) | 7-8, Table 1 |
| Interventions | 11a | Interventions for each group with sufficient detail to allow replication, including how and when they will be administered | 10-12 |
|  | 11b | Criteria for discontinuing or modifying allocated interventions for a given trial participant (eg, drug dose change in response to harms, participant request, or improving/worsening disease) | NA |
|  | 11c | Strategies to improve adherence to intervention protocols, and any procedures for monitoring adherence (eg, drug tablet return, laboratory tests) | 8 |
|  | 11d | Relevant concomitant care and interventions that are permitted or prohibited during the trial | 11-12 |
| Outcomes | 12 | Primary, secondary, and other outcomes, including the specific measurement variable (eg, systolic blood pressure), analysis metric (eg, change from baseline, final value, time to event), method of aggregation (eg, median, proportion), and time point for each outcome. Explanation of the clinical relevance of chosen efficacy and harm outcomes is strongly recommended | 12 |
| Participant timeline | 13 | Time schedule of enrolment, interventions (including any run-ins and washouts), assessments, and visits for participants. A schematic diagram is highly recommended (see Figure) | 8-10, Table 2 |
| Sample size | 14 | Estimated number of participants needed to achieve study objectives and how it was determined, including clinical and statistical assumptions supporting any sample size calculations | 16 |
| Recruitment | 15 | Strategies for achieving adequate participant enrolment to reach target sample size | 8 |

| **Methods: Assignment of interventions (for controlled trials)** | | |  |
| --- | --- | --- | --- |
| Allocation: |  |  |  |
| Sequence generation | 16a | Method of generating the allocation sequence (eg, computer-generated random numbers), and list of any factors for stratification. To reduce predictability of a random sequence, details of any planned restriction (eg, blocking) should be provided in a separate document that is unavailable to those who enrol participants or assign interventions | 9 |
| Allocation concealment mechanism | 16b | Mechanism of implementing the allocation sequence (eg, central telephone; sequentially numbered, opaque, sealed envelopes), describing any steps to conceal the sequence until interventions are assigned | 9 |
| Implementation | 16c | Who will generate the allocation sequence, who will enrol participants, and who will assign participants to interventions | 9 |
| Blinding (masking) | 17a | Who will be blinded after assignment to interventions (eg, trial participants, care providers, outcome assessors, data analysts), and how | 9, 12 |
|  | 17b | If blinded, circumstances under which unblinding is permissible, and procedure for revealing a participant’s allocated intervention during the trial | NA |
| **Methods: Data collection, management, and analysis** | | |  |
| Data collection methods | 18a | Plans for assessment and collection of outcome, baseline, and other trial data, including any related processes to promote data quality (eg, duplicate measurements, training of assessors) and a description of study instruments (eg, questionnaires, laboratory tests) along with their reliability and validity, if known. Reference to where data collection forms can be found, if not in the protocol | Table 2, Table 6, Appendix C |
|  | 18b | Plans to promote participant retention and complete follow-up, including list of any outcome data to be collected for participants who discontinue or deviate from intervention protocols | 8 |
| Data management | 19 | Plans for data entry, coding, security, and storage, including any related processes to promote data quality (eg, double data entry; range checks for data values). Reference to where details of data management procedures can be found, if not in the protocol | 16 |
| Statistical methods | 20a | Statistical methods for analysing primary and secondary outcomes. Reference to where other details of the statistical analysis plan can be found, if not in the protocol | 16-17 |
|  | 20b | Methods for any additional analyses (eg, subgroup and adjusted analyses) | 17 |
|  | 20c | Definition of analysis population relating to protocol non-adherence (eg, as randomised analysis), and any statistical methods to handle missing data (eg, multiple imputation) | 17 |
| **Methods: Monitoring** | | |  |
| Data monitoring | 21a | Composition of data monitoring committee (DMC); summary of its role and reporting structure; statement of whether it is independent from the sponsor and competing interests; and reference to where further details about its charter can be found, if not in the protocol. Alternatively, an explanation of why a DMC is not needed | NA |
|  | 21b | Description of any interim analyses and stopping guidelines, including who will have access to these interim results and make the final decision to terminate the trial | NA |
| Harms | 22 | Plans for collecting, assessing, reporting, and managing solicited and spontaneously reported adverse events and other unintended effects of trial interventions or trial conduct | 15 |
| Auditing | 23 | Frequency and procedures for auditing trial conduct, if any, and whether the process will be independent from investigators and the sponsor | NA |
| Ethics and dissemination | | |  |
| Research ethics approval | 24 | Plans for seeking research ethics committee/institutional review board (REC/IRB) approval | 7 |
| Protocol amendments | 25 | Plans for communicating important protocol modifications (eg, changes to eligibility criteria, outcomes, analyses) to relevant parties (eg, investigators, REC/IRBs, trial participants, trial registries, journals, regulators) | NA |
| Consent or assent | 26a | Who will obtain informed consent or assent from potential trial participants or authorised surrogates, and how (see Item 32) | 8 |
|  | 26b | Additional consent provisions for collection and use of participant data and biological specimens in ancillary studies, if applicable | NA |
| Confidentiality | 27 | How personal information about potential and enrolled participants will be collected, shared, and maintained in order to protect confidentiality before, during, and after the trial | 16 |
| Declaration of interests | 28 | Financial and other competing interests for principal investigators for the overall trial and each study site | 22 |
| Access to data | 29 | Statement of who will have access to the final trial dataset, and disclosure of contractual agreements that limit such access for investigators | 16 |
| Ancillary and post-trial care | 30 | Provisions, if any, for ancillary and post-trial care, and for compensation to those who suffer harm from trial participation | 8 |
| Dissemination policy | 31a | Plans for investigators and sponsor to communicate trial results to participants, healthcare professionals, the public, and other relevant groups (eg, via publication, reporting in results databases, or other data sharing arrangements), including any publication restrictions | 16 |
|  | 31b | Authorship eligibility guidelines and any intended use of professional writers | 22,23 |
|  | 31c | Plans, if any, for granting public access to the full protocol, participant-level dataset, and statistical code | 16 |
| Appendices |  |  |  |
| Informed consent materials | 32 | Model consent form and other related documentation given to participants and authorised surrogates | Appendix C |
| Biological specimens | 33 | Plans for collection, laboratory evaluation, and storage of biological specimens for genetic or molecular analysis in the current trial and for future use in ancillary studies, if applicable | NA |

*It is strongly recommended that this checklist be read in conjunction with the SPIRIT 2013 Explanation & Elaboration for important clarification on the items. Amendments to the protocol should be tracked and dated. The SPIRIT checklist is copyrighted by the SPIRIT Group under the Creative Commons “[Attribution-NonCommercial-NoDerivs 3.0 Unported](http://www.creativecommons.org/licenses/by-nc-nd/3.0/)” license.

**Appendix C: Forms and Questionnaires.**

**1. Informed consent**

**Patient Consent Form**

**(art. 13 D.Igs. 196/2003)**

**TITLE OF THE STUDY: The Italian Diabetes and Exercise Study 2 (IDES-2): a long-term behavioral intervention for adoption and maintenance of a physically active lifestyle**

**IDENTIFICATION OF THE STUDY**

Dear Patient,

The study in which your Diabetologist takes part has as its principal aim to demonstrate that exercise counseling is able to increase compliance to physical activity in the long term (3 years). The research includes the involvement of 300 patients with type 2 diabetes, it will be carried out in Rome and Province, supported by the Metabolic Fitness Association , a non-profit organization

**PROTECTION OF PERSONAL DATA:**

Any information that you provide, the collection and processing of which is indispensable to achieving the objectives of this study, will be treated with methods that guarantee absolute privacy, confidentiality and security in compliance with the standards for the protection of personal data and the right to privacy (Law 31 December 1996 n. 675 and subsequent amendments / additions).

You will be identified by a code and any clinical information concerning you will not be disclosed without prior written permission. The data collected will be made up of initials, date of birth, sex and otherwise sensitive clinical data such as disclosing your state of health.

As a person interested in the processing of your personal data, you will have full access to your information held by your doctor with the authority to exercise the rights of cancellation, transformation, integration, updating, rectification and block within the limits provided for by Article 13 of Law 675/96 regarding the protection of personal data. You will not be billed for exams, whose results will be communicated to your family doctor.

Your participation in this study is completely free and your doctor reserves the right to decide not to enroll you as he/she sees appropriate.

**BENEFITS OF THE STUDY:**

By agreeing to participate in this study, your doctor will be aware of the presence of cardiovascular risk factors to be monitored. Also the knowledge that will be acquired through your participation will be useful both for you and for other patients.

If you have any questions or clarifications, please do not hesitate to speak with your doctor. Please sign and date the following page to confirm that you have read all the information provided and have fully understood the purpose of this study and you freely give consent for the treatment of your personal data. The original of this Personal Consent Form will be deposited in the Archives of the Metabolic Fitness Association. You may also request a copy of your Personal Consent Form.

**Consent to the treatment of personal data**

**TITLE OF THE STUDY: The Italian Diabetes and Exercise Study 2 (IDES-2): a long-term behavioral intervention for adoption and maintenance of a physically active lifestyle**

I declare to accept the proposal to participate in the study described in this document. I understood all the information and explanations that were given to me and I had enough time to consider whether to make available, for the purposes of this study, clinical data concerning my person. I consent, in accordance with law 675/96 regarding the protection of personal data, so that clinical data concerning me and concerning the study in question are made available and published in the scientific literature. I agree in particular that the processing of my personal data, including those relating to health, is made for the purposes of research in the limits and in the manner shown to me in this Information and Consent Form.

**Signature of the patient _____________________ date____________________**

**Surname and name of the patient _____________________________________**

**Medical investigator's signature ____________________ date_____________________**

**Medical Investigator ‘ name _____________________________________**

2. Self-reported questionnaire for MS symptoms.

| **SHOULDER** | |
| --- | --- |
| **1** | Do you have pain during rotation of the arm ? |
| **2** | Are you awakened by pain during the night ? |
| **3** | Do you have pain on reaching objects above the head ? |
| **4** | Do you have pain on lifting objects ? |
| **5** | Do you have pain or soreness upon awakening that passes later on during the day ? |
| **6** | Have you taken anti-inflammation drugs or pain-killers ? |
| **ARM** | |
| **7** | Do you feel that you have less strength ? |
| **8** | Dou you feel that one arm is weaker than the other ? |
| **9** | Do you have pain at the maximum extension of the forearm ?. |
| **ELBOW** | |
| **10** | Do you have pain on lifting an object ? |
| **11** | Do you have pain on hitting against a rigid object ? |
| **12** | Have you taken anti-inflammation drugs or pain-killers ? |
| **WRIST** | |
| **13** | Do you have pain on lifting an object ? |
| **14** | Do you have pain on hitting against a rigid object ? |
| **15** | Have you taken anti-inflammation drugs or pain-killers ? |
| **HAND:** Do you feel "pins and needles" ? If so, in which finger ? | |
| **16** | I |
| **17** | II |
| **18** | III |
| **19** | IV |
| **20** | V |
| **SPINE: THORACO-CERVICAL** | |
| **21** | Do you have pain/tenderness/ pins & needles on turning your head from side to side ? |
| **22** | Do you often have pain or headache or heaviness of the head or neck ? |
| **23** | Do you have pain between the shoulder blades ? |
| **24** | Do you feel it necessary to move your head from side to side to get moving and feel ? |
| **25** | Do you have episodes of painful sudden acute stiffness of the neck ? |
| **26** | Have you taken anti-inflammation drugs or pain-killers ? |
| **SPINE: LUMBO-SACRAL** | |
| **27** | Do you have pain on bending to tie your shoe laces ? |
| **28** | Do you have any back-pain on turning left or right ? |
| **29** | Do you have a feeling of heaviness in your back on standing for long hours ? |
| **30** | Do you have bothersome feeling when sitting still ? Do you have to get up ? |
| **31** | Did you have one episode of sudden intense back pain that leaves you unable to move ? |
| **32** | Have you taken anti-inflammation drugs or pain-killers ? |
| **HIP** | |
| **33** | Do you have pain on crossing your legs ? |
| **34** | Do you have any pain when opening your legs to the maximum ? |
| **35** | Do you often have pain from your buttocks along the length of the leg down to your ankles ? |
| **36** | Have you taken a single dose of anti-inflammatory drugs or pain-killers ? |
| **KNEE** | |
| **37** | Do you have pain in the knee in the act of sitting down or getting up ? |
| **38** | Do you have pain in your knee after having walked a lot ? |
| **39** | Is your knee often swollen at the end of the day ? |
| **40** | Do you have pain in the "good" knee ? |
| **41** | Do you have pain or a bothersome feeling as you a kneel down ? |
| **42** | When lying in bed, do you feel the need to move your legs, ones or more than once ? |
| **43** | Have you taken a single dose of anti-inflammatory drugs or pain-killers ? |
| **FOOT** | |
| **44** | Do you often feel a sensation of pins and needles that runs down to one or more toes ? |
| **45** | Do you have any difficulty in standing on your toes ? |
| **46** | Do you have any pain in your foot after walking for a long time ? |
| **47** | Do you have pain on taking the first step in the morning ? |
| **48** | Do you have any difficulty or pain when putting on stiff orthopedic shoes ? |
| **49** | Do you have pain under the heel when walking a lot ? |
| **50** | Have you taken a single dose of anti-inflammatory drugs or pain-killers ? |

**3. WHO-5 Well-Being Index (1998 version)**

Please indicate for each of the five statements which is closest to how you have been feeling over the last two weeks.

Notice that higher numbers mean better well-being.

Example: If you have felt cheerful and in good spirits more than half of the time during the last two weeks, put a tick in the box with the number 3 in the upper right corner.

| **#** | **Over the last two weeks** | **All of the time** | **Most of the time** | **More than half of the time** | **Less than half of the time** | **Some of the time** | **At no time** |
| --- | --- | --- | --- | --- | --- | --- | --- |
| **1** | **I have felt cheerful and in good spirits** | 🞎 5 | 🞎 4 | 🞎 3 | 🞎 2 | 🞎 1 | 🞎 0 |
| **2** | **I have felt calm and relaxed** | 🞎 5 | 🞎 4 | 🞎 3 | 🞎 2 | 🞎 1 | 🞎 0 |
| **3** | **I have felt active and vigorous** | 🞎 5 | 🞎 4 | 🞎 3 | 🞎 2 | 🞎 1 | 🞎 0 |
| **4** | **I woke up feeling fresh and rested** | 🞎 5 | 🞎 4 | 🞎 3 | 🞎 2 | 🞎 1 | 🞎 0 |
| **5** | **My daily life has been filled with things that interest me** | 🞎 5 | 🞎 4 | 🞎 3 | 🞎 2 | 🞎 1 | 🞎 0 |

**Scoring:**

The raw score is calculated by totalling the figures of the five answers. The raw score ranges from 0 to 25, 0 representing worst possible and 25 representing best possible quality of life.

To obtain a percentage score ranging from 0 to 100, the raw score is multiplied by 4. A percentage score of 0 represents worst possible, whereas a score of 100 represents best possible quality of life.

**Interpretation:**

It is recommended to administer the Major Depression (ICD-10) Inventory if the raw score is below 13 or if the patient has answered 0 to 1 to any of the five items. A score below 13 indicates poor wellbeing and is an indication for testing for depression under ICD-10.

**Monitoring change:**

In order to monitor possible changes in wellbeing, the percentage score is used. A 10% difference indicates a significant change (ref. John Ware, 1995).

**4. SF-36 health survey**

**SF-36 QUESTIONNAIRE**

**Name: _______________________ Ref. Dr: _______________________ Date: __________**

**ID#: _______________ Age: _______ Gender: M / F**

Please answer the 36 questions of the **Health Survey** completely, honestly, and without interruptions.

**1. GENERAL HEALTH:**

**In general, would you say your health is:**

🞎 Excellent 🞎 Very Good 🞎 Good 🞎 Fair 🞎 Poor

**Compared to one year ago, how would you rate your health in general now?**

🞎 Much better now than one year ago

🞎 Somewhat better now than one year ago

🞎 About the same

🞎 Somewhat worse now than one year ago

🞎 Much worse than one year ago

**2. LIMITATIONS OF ACTIVITIES:**

The following items are about activities you might do during a typical day. Does your health now limit you in these activities? If so, how much?

**Vigorous activities, such as running, lifting heavy objects, participating in strenuous sports.**

🞎 Yes, limited a lot 🞎 Yes, Limited a Little 🞎 No, Not Limited at all

**Moderate activities, such as moving a table, pushing a vacuum cleaner, bowling, or playing golf**

🞎 Yes, limited a lot 🞎 Yes, Limited a Little 🞎 No, Not Limited at all

**Lifting or carrying groceries**

🞎 Yes, limited a lot 🞎 Yes, Limited a Little 🞎 No, Not Limited at all

**Climbing several flights of stairs**

🞎 Yes, limited a lot 🞎 Yes, Limited a Little 🞎 No, Not Limited at all

**Climbing one flight of stairs**

🞎 Yes, limited a lot 🞎 Yes, Limited a Little 🞎 No, Not Limited at all

**Bending, kneeling, or stooping**

🞎 Yes, limited a lot 🞎 Yes, Limited a Little 🞎 No, Not Limited at all

**Walking more than a mile**

🞎 Yes, limited a lot 🞎 Yes, Limited a Little 🞎 No, Not Limited at all

**Walking several blocks**

🞎 Yes, limited a lot 🞎 Yes, Limited a Little 🞎 No, Not Limited at all

**Walking one block**

🞎 Yes, limited a lot 🞎 Yes, Limited a Little 🞎 No, Not Limited at all

**Bathing or dressing yourself**

🞎 Yes, limited a lot 🞎 Yes, Limited a Little 🞎 No, Not Limited at all

**3. PHYSICAL HEALTH PROBLEMS:**

During the past 4 weeks, have you had any of the following problems with your work or other regular daily activities as a result of your physical health?

**Cut down the amount of time you spent on work or other activities**

🞎 Yes 🞎 No

**Accomplished less than you would like**

🞎 Yes 🞎 No

**Were limited in the kind of work or other activities**

🞎 Yes 🞎 No

**Had difficulty performing the work or other activities (for example, it took extra effort)**

🞎 Yes 🞎 No

**4. EMOTIONAL HEALTH PROBLEMS:**

During the past 4 weeks, have you had any of the following problems with your work or other regular daily activities as a result of any emotional problems (such as feeling depressed or anxious)?

**Cut down the amount of time you spent on work or other activities**

🞎 Yes 🞎 No

**Accomplished less than you would like**

🞎 Yes 🞎 No

**Didn't do work or other activities as carefully as usual**

🞎 Yes 🞎 No

**5. SOCIAL ACTIVITIES:**

**Emotional problems interfered with your normal social activities with family, friends, neighbors, or groups?**

🞎 Not at all 🞎 Slightly 🞎 Moderately 🞎 Severe 🞎 Very Severe

**6. PAIN:**

**How much bodily pain have you had during the past 4 weeks?**

🞎 None 🞎 Very Mild 🞎 Mild 🞎 Moderate 🞎 Severe 🞎 Very Severe

**During the past 4 weeks, how much did pain interfere with your normal work (including both work outside the home and housework)?**

🞎 Not at all 🞎 A little bit 🞎 Moderately 🞎 Quite a bit 🞎 Extremely

**7. ENERGY AND EMOTIONS:**

These questions are about how you feel and how things have been with you during the last 4 weeks. For each question, please give the answer that comes closest to the way you have been feeling.

**Did you feel full of pep?**

🞎 All of the time

🞎 Most of the time

🞎 A good Bit of the Time

🞎 Some of the time

🞎 A little bit of the time

🞎 None of the Time

**Have you been a very nervous person?**

🞎 All of the time

🞎 Most of the time

🞎 A good Bit of the Time

🞎 Some of the time

🞎 A little bit of the time

🞎 None of the Time

**Have you felt so down in the dumps that nothing could cheer you up?**

🞎 All of the time

🞎 Most of the time

🞎 A good Bit of the Time

🞎 Some of the time

🞎 A little bit of the time

🞎 None of the Time

**Have you felt calm and peaceful?**

🞎 All of the time

🞎 Most of the time

🞎 A good Bit of the Time

🞎 Some of the time

🞎 A little bit of the time

🞎 None of the Time

**Did you have a lot of energy?**

🞎 All of the time

🞎 Most of the time

🞎 A good Bit of the Time

🞎 Some of the time

🞎 A little bit of the time

🞎 None of the Time

**Have you felt downhearted and blue?**

🞎 All of the time

🞎 Most of the time

🞎 A good Bit of the Time

🞎 Some of the time

🞎 A little bit of the time

🞎 None of the Time

**Did you feel worn out?**

🞎 All of the time

🞎 Most of the time

🞎 A good Bit of the Time

🞎 Some of the time

🞎 A little bit of the time

🞎 None of the Time

**Have you been a happy person?**

🞎 All of the time

🞎 Most of the time

🞎 A good Bit of the Time

🞎 Some of the time

🞎 A little bit of the time

🞎 None of the Time

**Did you feel tired?**

🞎 All of the time

🞎 Most of the time

🞎 A good Bit of the Time

🞎 Some of the time

🞎 A little bit of the time

🞎 None of the Time

**8. SOCIAL ACTIVITIES:**

**During the past 4 weeks, how much of the time has your physical health or emotional problems interfered with your social activities (like visiting with friends, relatives, etc.)?**

🞎 All of the time

🞎 Most of the time

🞎 Some of the time

🞎 A little bit of the time

🞎 None of the Time

**9. GENERAL HEALTH:**

**How true or false is each of the following statements for you?**

**I seem to get sick a little easier than other people**

🞎 Definitely true 🞎 Mostly true 🞎 Don't know 🞎 Mostly false 🞎 Definitely false

**I am as healthy as anybody I know**

🞎 Definitely true 🞎 Mostly true 🞎 Don't know 🞎 Mostly false 🞎 Definitely false

**I expect my health to get worse**

🞎 Definitely true 🞎 Mostly true 🞎 Don't know 🞎 Mostly false 🞎 Definitely false

**My health is excellent**

🞎 Definitely true 🞎 Mostly true 🞎 Don't know 🞎 Mostly false 🞎 Definitely false

**Appendix D: World Health Organization Trial Registration Data Set**

1. **Primary Registry and Trial Identifying Number:** ClinicalTrials.gov; NCT01600937; URL <https://clinicaltrials.gov/ct2/show/NCT01600937>.
2. **Date of Registration in Primary Registry:** October 10, 2012
3. **Secondary Identifying Numbers:** NA.
4. **Source(s) of Monetary or Material Support:** Metabolic Fitness Association O.N.L.U.S., Via Nomentana, 27 - 00016 Monterotondo Scalo, Rome, Italy; Phone +390690080260; Fax: +390690080235; e-mail: [info@metabolicfitness.it](mailto:info@metabolicfitness.it).
5. **Primary Sponsor:** Metabolic Fitness Association O.N.L.U.S., Via Nomentana, 27 - 00016 Monterotondo Scalo, Rome, Italy; Phone +390690080260; Fax: +390690080235; e-mail: [info@metabolicfitness.it](mailto:info@metabolicfitness.it).
6. **Secondary Sponsor(s):** NA.
7. **Contact for Public Queries: Stefano Balducci, MD,** Metabolic Fitness Association O.N.L.U.S., Via Nomentana, 27 - 00016 Monterotondo Scalo, Rome, Italy; Phone +390690080260; Fax: +390690080235; e-mail: [sbalducci@esinet.it](mailto:sbalducci@esinet.it).
8. **Contact for Scientific Queries: Giuseppe Pugliese,** , M.D., Ph.D., Department of Clinical and Molecular Medicine, “La Sapienza” University of Rome, Via di Grottarossa, 1035-1039 - 00189 Rome, Italy; Phone: +39-0633775440; Fax: +39-0633776327; E-mail: [giuseppe.pugliese@uniroma1.it](mailto:giuseppe.pugliese@uniroma1.it).
9. **Public Title:** The Italian Diabetes and Exercise Study 2 (IDES-2): a long-term behavioral intervention for adoption and maintenance of a physically active lifestyle
10. **Scientific Title:** The Italian Diabetes and Exercise Study 2 (IDES-2): a long-term behavioral intervention for adoption and maintenance of a physically active lifestyle
11. **Countries of Recruitment:** Italy.
12. **Health Condition(s) or Problem(s) Studied:** type 2 diabetes mellitus.
13. **Intervention:**
14. Intervention arm

- Name: Aggregated behavior change techniques.
- Description: one individual theoretical counseling session, held by a physician (diabetologist) plus eight twice-a-week individual theoretical and practical exercise counseling sessions, held by a certified exercise specialist, once-a-year for three years, on top of standard care.

1. Control arm: general physician recommendations for increasing the amount of daily PA and decreasing the SED-time on top of standard care.
2. **Key Inclusion and Exclusion Criteria**
3. Inclusion criteria: known T2DM (defined by the ADA criteria) of at least 1-year duration. Additional requirements are age 40-80 years, BMI 27-40 kg/m^2^, sedentary lifestyle (i.e. more than 8 hours/day spent in any waking behavior characterized by an energy expenditure ≤1.5 METs while in a sitting or reclining posture) and physically inactivity (i.e. insufficient amounts of PA according to current guidelines) from at least 6 months, ability to walk 1.6 Km without assistance, and eligibility after cardiologic evaluation.
4. Exclusion criteria: unable or unwilling to give informed consent or communicate with local study staff; current diagnosis of psychiatric disorder or hospitalization for depression in the past six months; self-reported alcohol or substance abuse within the past twelve months, self-reported inability to walk two blocks; musculoskeletal disorders or deformities that may interfere with participation in the intervention; history of central nervous dysfunction such as hemiparesis, myelopathies, cerebral ataxia; clinical evidence of vestibular dysfunction; postural hypotension defined as a fall in BP when changing position of >20 mmHg (systole) or >10 mmHg (diastole); currently pregnant or nursing; cancer requiring treatment in the past five years, except for cancers that have clearly been cured or in the opinion of the investigator carry an excellent prognosis (e.g., stage 1 cervical cancer); chronic obstructive pulmonary disease; end-stage liver disease; chronic diabetic complications (recent major acute cardiovascular event, including heart attack, stroke/transient ischemic attack(s), revascularization procedure, or participation in a cardiac rehabilitation program within the past three months; pre-proliferative and proliferative retinopathy; macroalbuminuria and/or eGFR < 45 ml/min/1.73 m^2^; severe motor and sensory neuropathy; diabetic foot with history of ulcer); cardiovascular disease at cardiologic examination (history of cardiac arrest; history of pulmonary embolism in the past six months; unstable angina pectoris or angina pectoris at rest; resting HR <45 beats/min or >100 beats/min; complex ventricular arrhythmia at rest or with exercise; uncontrolled atrial fibrillation (HR >100 beats/min); NYHA Class III or IV congestive heart failure; acute myocarditis, pericarditis or hypertrophic myocardiopathy; left bundle branch block or cardiac pacemaker); conditions not specifically mentioned above at the discretion of the clinical site.
5. **Study Type**
   1. Type of study: interventional.
   2. Study design:
      - Method of allocation: randomized
      - Masking: no
      - Assignment: parallel
      - Purpose: testing the efficacy of a behavior change strategy in increasing total daily PA and reducing sedentary (SED)-time in patients with T2DM
6. Phase: NA
7. Allocation concealment mechanism and sequence generation: centralized randomization stratified by center and, within each center, by age and type of diabetes treatment (non-insulin versus insulin therapy, using a permuted-block randomization software which randomly varies the block size.
8. **Date of First Enrollment:** January 7, 2014.
9. **Target Sample Size:** 300
10. **Recruitment Status: recruiting.**
11. **Primary Outcome(s)**
    - Name: physical activity and sedentary time;
    - Method of measurement: accelerometer and daily diary;
    - Time points: every 4 months for 36 months.
12. **Key Secondary Outcomes**
    1. Name: physical fitness; methods of measurement: maximal treadmill exercise test, isometric muscle strength test; bending test; time points: every 12 months for 36 months.
    2. Name: modifiable cardiovascular risk factors; methods of measurement: clinical and biochemical testing; time points: every 4 months for 36 months.
    3. Name: musculoskeletal disturbances; method of measurement: self-report questionnaire; time points: at 4, 12, 16, 24, 28 and 36 months.
    4. Name: well-being/depression; method of measurement: WHO-5 Well-being Index; time points: at 4, 12, 16, 24, 28 and 36 months.
    5. Name: health-related quality of life; method of measurement: SF-36 health survey; time points: at 4, 12, 16, 24, 28 and 36 months.
